# Safety of Cultivated Limbal Epithelial Stem Cell Transplantation for Human Corneal Regeneration

**DOI:** 10.1155/2017/6978253

**Published:** 2017-03-30

**Authors:** J. Behaegel, S. Ní Dhubhghaill, C. Koppen, N. Zakaria

**Affiliations:** ^1^Faculty of Medicine and Health Sciences, Department of Ophthalmology, Visual Optics and Visual Rehabilitation, University of Antwerp, Campus Drie Eiken, T building, T4-Ophthalmology, Universiteitsplein 1, 2610 Antwerp, Belgium; ^2^Center for Cell Therapy and Regenerative Medicine, Antwerp University Hospital, CCRG-Oogheelkunde, Wilrijkstraat 10, 2650 Edegem, Belgium; ^3^Department of Ophthalmology, Brussels University Hospital, Dienst Oogheelkunde, Laarbeeklaan 101, 1090 Jette, Belgium; ^4^Department of Ophthalmology, Antwerp University Hospital, Dienst Oogheelkunde, Wilrijkstraat 10, 2650 Edegem, Belgium

## Abstract

Ex vivo cultivated limbal stem cell transplantation is a promising technique for the treatment of limbal stem cell deficiency. While the results of the clinical trials have been extensively reported since the introduction of the technique in 1997, little has been reported regarding the potential health risks associated with production processes and transplantation techniques. Culture procedures require the use of animal and/or human-derived products, which carry the potential of introducing toxic or infectious agents through contamination with known or unknown additives. Protocols vary widely, and the risks depend on the local institutional methods. Good manufacturing practice and xeno-free culture protocols could reduce potential health risks but are not yet a common practice worldwide. In this review, we focus on the safety of both autologous- and allogeneic-cultivated limbal stem cell transplantation, with respect to culture processes, surgical approaches, and postoperative strategies.

## 1. Introduction

In recent years, stem cell research advances have made revolutionary changes in medicine and resulted substantial benefits to patients suffering a wide range of diseases and injuries. Ophthalmology in particular has benefited from stem cell-based regenerative treatment, and further cell-based research is still promising for the future.

Limbal epithelial transplantation is a prime example of these cell-based therapies that have been used successfully in patients suffering from limbal stem cell deficiency (LSCD). The goal in LSCD management is to restore the limbal microenvironment and for the cornea to regain a corneal epithelial phenotype by transplantation of limbal stem cells.

The earliest treatments for limbal stem cell transplantations included keratolimbal lamellar allograft (KLAL), conjunctival-limbal autografts (CLAU), and living-related conjunctival-limbal allografts (lr-CLAL), all of which required large sections of limbal donor tissue. In 1997, the results of the first cultivated limbal stem cell transplantation (CLET) were reported [1]. This technique required only a small donor biopsy, reducing both the amount of tissue harvested and risks to the donor eye.

Moreover, the dose and the duration of systemic immunosuppression could be significantly reduced as cultured allografts once transplanted showed limited long-term survival [2–4]. While it has advantages, the ex vivo culture protocol does introduce new risks, namely those related to the culture processing methods. This includes potential contamination with known or unknown infectious agents introduced by the use of animal and/or human tissue. Furthermore, good manufacturing practice (GMP), rigid traceability, and careful operative techniques are also key elements that should be considered when determining the safety of a stem cell therapy.

This review focuses on the different manufacturing methods, surgical techniques, and postoperative strategies of cultivated limbal stem cell transplantation. Here, we present an overview of the literature in the field over the past 10 years.

## 2. Method of Literature Search

Literature search was conducted on the electronic database “Pubmed” with the key words “cultivated limbal stem cell transplantation.” Reference lists were scanned in order to identify any additional trials. The search was performed in March 2016 and restricted to English language reports and to articles published over the last 10 years, starting from January 2006. Original studies and case reports including at least 1 case of a human autologous- or allogeneic-cultivated limbal stem cell transplantation were included. When trials included more sources of stem cell tissue for cultivation, the data were filtered to limbal tissue only. Multiple trial reports from the same groups were not excluded. In total, 32 human clinical studies were included. Many studies published data and culture details systematically, but this was not the case for all trials, and missing data was recorded as such.

## 3. Origin of the Cells

Both autologous and allogeneic sources of limbal epithelial stem cells have been used in clinical trials.

Autologous cells are preferred as they have no risk of immunoreactivity and require no systemic immunosuppression. This is not possible in cases of bilateral disease, and options are limited to tissue donation from deceased or living-related donors. Autologous limbal tissue was used for culture in 20 of the 32 (62,5%) reviewed studies [5–24], allogeneic donor material was used in 3 (9,4%) [25–27], and both were used in 9 (28,1%) studies [28–36]. Of the 12 groups including allogeneic transplantations, one group used living-related donor material only for biopsy harvesting [26], 6 trials used cadaveric material only [25, 27, 28, 31, 34, 36], and in 5 trials, both cadaveric and living-related sources were used [29, 30, 32, 33, 35]. Details regarding to in- or exclusion of the cadaveric donor eyes were lacking in almost all trials. Only one group provided details on the age limit (<60 y) for inclusion of cadaveric donor sources [36]. Details of the origin of the cells and culture techniques are described in Table 1.

The benefit of preoperative HLA typing is currently unclear. Only two papers reported HLA typing prior to biopsy harvesting from allogeneic donors [26, 35]. One was a case report where biopsy was taken from an HLA-identical living-related donor who had also donated peripheral blood stem cells to treat an acute myeloid leukemia [26]. The patient showed a successful ocular surface reconstruction after a follow-up of 31 months, though this may be because the transplanted immune system was identical to the graft. The other paper included three allogeneic donors, two living-related donors, and one cadaveric donor [35]. A three-loci match of at least 50% (HLA-A, HLA-B, and HLA-DR) was required of the living-related donors. HLA matching of the cadaveric donor was not performed. Both living-related transplantations achieved successful results while the unmatched cadaveric transplant failed. In both of the articles, no side effects directly related to immunoreactivity were reported. Requiring a graft to be HLA-matched would incur a higher cost and drastically reduce the cadaveric donor pool so an HLA-matched advantage would have to be unequivocal.

In the literature, only one group specifically mentioned side effects directly related to immunoreactivity [27]. In this trial, immune rejection occurred in 10 of 42 eyes (23,8%) following allogeneic CLET. In 7 (70%) of the patients, this led to redness, irritation, photophobia, and decreased visual acuity. Additionally, they stated that a delayed recognition could potentially result in a worsening of the corneal opacification and neovascularization. Definitive diagnosis of epithelial rejection is difficult as new symptoms accompanied by epithelial breakdown are hard to attribute directly to an immune rejection rather than primary failure of the stem cell treatment and conjunctivalization. It is possible though, when compared with direct tissue transfer, that CLET may have a reduced risk of graft rejection as antigen-presenting macrophages do not survive the culturing process [37].

## 4. Culture Process

### 4.1. Feeder Layers

Many of the published cell culture protocols depend on the support of a 3T3 cell feeder layer to nurture the graft. The murine fibroblast cells within the 3T3 layer allow epithelial cells to spread and form uniform layers. The cells are either irradiated or treated by mitomycin C to inactivate growth. In the literature, 11 culture protocols required use of murine 3T3 cells during culture processing [7, 10, 11, 13, 23–25, 27–30]. Two of them also described the use of a feeder-free culture method [28, 30]. In most of the studies, the method of inactivation was reported. Five studies used mitomycin C for growth inactivation [7, 25, 27, 28, 30], and 5 stated use of irradiation [10, 11, 13, 23, 24]. Both inactivation methods inhibit DNA replication and are considered to be qualitatively equivalent [38].

Despite this inactivation method, there is still an exposure to animal material during the culture period. This implies that the use of a 3T3 cell feeder layer carries risks in terms of rejection, microchimerism, and infection with virus or prion agents [38–40] though to date, no such events have been reported. Furthermore, cells cultured under xeno-contaminated conditions can present a nonhuman sialic acid, which has been shown to be immunogenic to humans [41, 42]. These results have given rise to concern of analogous risks in CLET.

Some clinical trials have pivoted away from the use of xenogenic feeder cells with the goal of generating a “xeno-free” protocol and product. The 3T3 layers have been replaced by human-derived feeder layers [43–45], or not at all in feeder-free culture methods [5, 6, 8, 14–22, 26, 28, 31–34, 36]. Feeder layers from human origin also carry the risk of contamination by human viral and nonviral infectious agents and prions, which raises similar questions concerning its safety profile. In this respect, feeder-free culture protocols are theoretically least harmful and most ideal in the development of the safest culture protocol.

### 4.2. Culture Serum

Fetal bovine serum (FBS) has been used extensively to nurture limbal stem cell cultures and provides factors required for cell attachment, growth, and proliferation [46]. The serum is harvested from bovine foetuses taken from pregnant cows during slaughter [47]. Irradiation of FBS is frequently used to eliminate live virus, but no process can be guaranteed as fully effective over time [48]. In a study of 26 commercially available FBS products, all samples contained at least one species of bovine pestivirus by RT-PCR [48]. Fifteen samples tested positive for a putative pestivirus, the “HoBi-like” virus that appears to be related to bovine viral diarrhoea virus (BVDV) [49]. The origin of this HoBi-like is unknown, but it may have originated in South-America and introduced into Europe and South-East Asia through biological products like FBS, vaccines, and semen [49]. If this is the case, it suggests possible contamination, mislabelling, or mixing of sera from different regions during the processing and packaging process of FBS. These findings have implications for patient safety in the use of FBS and strengthen the arguments for a xenogenic free or a serum-free culture media.

Human serum has been used to replace the need for FBS in the culture of a variety of epithelial cell types. In cases where human autologous serum can be used, the serum is derived from the donated blood of the patient to be treated with the cell product. This exposes the patient only to their own blood products, reducing the potential risks of disease transmission and immune rejection [50]. It does however require that the patient be medically stable to donate the blood required. In addition, patients with blood borne diseases such as HIV or hepatitis are not eligible to give their own serum as knowingly culturing with contaminated product is too high a risk, not only for the cell product but also for the laboratory staff and other cell cultures. Another option is to use pooled human serum which is both simpler to use and more consistent than autologous serum. However, similarly as in the use of human-derived feeder layers, the theoretical risk of contamination by infectious agents and prions also exists when using human-derived serum. Clinical grade blood products are screened to the highest standards, and the introduction of nucleic acid amplification testing (NAT) has further reduced risks of HIV, hepatitis B, and hepatitis C infections [51, 52]. Although these well-known viruses are considered as being under control, their absence cannot be completely guaranteed and the discovery of novel pathogens in human-derived blood products [53] raises issues of new infection risks in the future.

Efforts have been made to investigate xenogenic- and serum-free culture protocols [54–56], but according to our knowledge, they have not yet been used in human clinical trials. Over the last 10 years, the use of human serum is gaining popularity compared to FBS. Since 2006, 16 studies reported the use of human serum only for clinical use [6–9, 12, 15, 16, 18, 19, 22, 26, 28, 30, 32, 34, 35]. In the majority of these trials, the source was autologous in origin with a concentration varying between 3 to 5%, while in one of the trials a clinical grade AB serum was used [35]. Eleven studies reported the use of FBS [11, 13, 14, 20, 23–25, 27, 31, 33, 36], and 4 studies included both types of serum or switched from FBS to AS [5, 17, 21, 29]. One study did not describe their use of serum in their protocol [10]. There were no reported side effects related to the serum choice.

### 4.3. Scaffolds

Human amniotic membrane (HAM) is the innermost layer of the placenta and has been extensively used in the treatment of ocular surface pathologies [57]. The substrate is purported to act as a surrogate environmental stem cell niche [58], and its biological constituents are thought to be responsible for its beneficial properties [57]. The membrane has an immunomodulatory effect [59], which explains why tissue rejection is not observed in its clinical use. Various methods have been used to preserve amniotic membranes including hypothermic (“fresh”) storage, freezing, and freeze drying of the HAM. Despite its many advantages, its clinical use also carries a theoretical risk of disease transmission since HAMs are always allogeneic in nature. In Western countries, strict legislation stipulates HIV, hepatitis B and C, and HTLV tests on the donor serum at the time of procuring the membrane [57]. Frozen storage of the tissue permits repeat blood testing of the donor 6 months following donation [60]. The use of frozen HAM therefore provides an extra level of reliability and security over the use of fresh amnion [60, 61]. In developing countries, fresh-unpreserved membranes are more commonly used. The short interval between procurement and use, however, provides no time for serological retesting of the donor. Even when serological tests can be performed, they do not exclude all possible risks of disease transmission with unknown pathogens or pathogens for which no tests are available such as Creutzfeldt-Jakob disease [61].

Currently, there are no published reports of communicable disease transmission from amniotic membrane transplantation. One report exists of a sterile hypopyon after repeated transplantation of human amniotic membrane on cornea surface, probably related to a localized immunoreaction [62]. Twenty-five of the 32 clinical trials reported (78,1%) used human amniotic membrane as a culture substrate [5–7, 9, 12, 14–21, 25–36]. No events of disease transmission related to the use of HAM were reported.

A number of alternative cell carriers including human- and animal-derived collagen [63–65], fibrin [10, 11, 13, 24], contact lenses [22, 66], human anterior lens capsules [67], and silk fibroin [68, 69] have been proposed to overcome disadvantages associated with HAM use such as disease transmission, variable tissue quality, and limited transparency. However, these alternatives also have their own drawbacks. Promising in this field is the Real Architecture For 3D Tissue (RAFT) technique that is able to recreate the three-dimensional (3D) limbal crypts in the surface of collagen-based tissue equivalents [70, 71].

In the recent literature, two human clinical trials used siloxane hydrogels contact lenses as a scaffold for cell expansion [8, 22]. In terms of safety, the lenses have the advantage being nonimmunogenic in nature and do not carry the risk transmission of tissue-derived pathogens. Although contact lens wear is known to be associated with limbal stem cell failure [72], this complication occurs only with long-term application. In these two trials, the contact lens was removed after a maximum of 22 days. Reported complications were limited to the occurrence of a small defect upon contact lens removal and rolling up of the contact lens following insertion on the eye.

Five trials reported the use of a fibrin matrix as a cell scaffold [10, 11, 13, 23, 24]. Fibrin derivatives are frequently used in ophthalmology as membranes or glue. Their safety properties are described further under “stabilization methods of the graft.”

Ideally, a tissue-engineered scaffold would provide a safer platform for cell therapy, and while there is in vitro work being done in this area, there have been no human clinical trials with novel scaffolds.

## 5. Surgical Procedure

### 5.1. Biopsy

The earliest limbal stem cell transplantation techniques required large sections of limbal donor tissue, which placed the donor eye at risk of developing stem cell deficiency. In CLET, only a small donor biopsy (1 × 2 mm or 2 × 2 mm) is harvested from the patient's fellow eye, from a living-related donor eye or from a living-unrelated donor eye. The smaller size of biopsy significantly reduces the risks of precipitating stem cell deficiency in the donor eye and offers the option of taking a second biopsy, if needed. There is no consensus on the safety threshold of the biopsy harvest, the maximum number of biopsies that may be taken or the total surface area that may be removed without compromising the donor eye.

While we suggest a maximum of 2 limbal biopsies from the same donor eye, we have not yet encountered stem cell deficiency in a donor eye. The number of tolerable biopsies may be greater. Two studies of the same group have harvested up to 3 autologous biopsies from separate sites of the same eye [8, 22]. There were no postoperative complications noted in the donor eyes. Basu et al. reported subconjunctival hemorrhage adjacent to the donor site in 13 of 50 (26%) eyes, but functionally this was insignificant [16].

### 5.2. Transplantation

Standardization of limbal stem cell transplantation is difficult since LSCD results from a broad spectrum of pathologies. Stem cell deficient eyes are rarely homogenous in corneal, tear film, and eyelid involvement. In general, the first step of the surgical graft procedure is a 360° peritomy followed by removal of the fibrovascular superficial pannus. There is no consensus about the amount of pannus that should be removed or the required residual corneal thickness after pannus removal. Aggressive excision may thin the cornea and can lead to perforation or wound dehiscence at the donor-recipient junction of a previous penetrating keratoplasty [73–75]. Conversely, a gentle excision may result in a residual fibrovascular barrier between the grafted stem cells and the cornea, impairing integration and reducing the visual potential [76].

In this review, 7 studies reported at least one perforation or a thinning/melting of the cornea after CLET, with a total of 11 eyes [14, 15, 17, 26, 30, 32, 35]. One of them reported 5 corneal melts in a total cohort of 200 patients [15]. All of the 5 patients being treatment failures. Limbal stem cell deficient eyes with delayed healing and a thin cornea are considered at higher risk for corneal melting and therefore should be monitored closely.

### 5.3. Stabilization Methods of the Graft

After removal of the fibrovascular pannus, the limbal stem cell graft is transferred to the eye and attached using either fibrin glue or suture fixation. In some protocols, mitomycin C is briefly applied, followed by irrigation. Suturing is the best-known stabilization method in limbal graft surgery and is performed by securing the scaffold at the level of, or just beyond the limbus. Sutures penetrate the limbal tissue and cause small regions of trauma, provoking localised inflammatory responses and a focus for infection. The local inflammation can act as a vasostimulatory agent and may stimulate new vessel growth [77].

More recently, fibrin glue is used as an alternative or additional method for the graft application. The use of fibrin glue can reduce operative time and postoperative inflammation, irritation, and pain [78, 79]. Other advantages include its flexibility and its biodegradable nature [80]. Similar to blood-derived cell culture products, a significant drawback to fibrin glue is the risk of transmitting serological diseases, since commercially available fibrin glue is made from pooled donor plasma [81]. Over the past 20 years, no case of infection transmitted through fibrin glue has been documented. Aprotinin is an antifibrinolytic agent, commonly used as an additive in fibrin sealant. Allergic and anaphylactic reactions to aprotinin, found in some fibrin glue preparations, have been described [82, 83]. The earlier use of bovine thrombin in fibrin sealant preparations has now been largely replaced by human thrombin.

Suture fixation was used in 23 of the 32 studies [5–7, 9, 10, 12–15, 18, 20, 23–33, 36] while 4 used the adhesive properties of fibrin [11, 16, 19, 35]. Both methods were used in 3 trials [17, 21, 34]. In addition, two trials described the use of siloxane hydrogels contact lenses which act both as a scaffold and application method for the transplanted stem cells [8, 22]. Their safety properties are described previously.

## 6. Postoperative Medication

Postoperative medication is administered either directly to the eye or systemically in order to avoid postoperative complications, reduce inflammation, and prevent graft rejection. In case of allogeneic transplantation, immunosuppressive agents are added in order to prevent graft rejection. Infection, prolonged inflammation, and allergic reactions are all possible complications of the described surgical methods. Nine papers reported infectious complications post-CLET [5, 15–17, 24, 30, 31, 34, 36]. In most papers, no details regarding possible cause of infection or time of occurrence are provided. It is therefore not possible to determine whether the reported complications are related to surgical, cultivation methods, or postop treatment. There have been no reported allergic reactions but one paper reported recurrent or persistent inflammation after surgery [23].

### 6.1. Local

The local postoperative treatment typically consists of topical antibiotics, steroids, and frequent lubricants. Antibiotic and anti-inflammatory agents make part of the standard postoperative care in corneal transplantation and will not be discussed in further detail. The use of preservative-free drops in CLET is recommended since preservatives can cause morphologic disruption of the corneal epithelium [84, 85]. Autologous serum drops have also shown efficacy in treating ocular surface disorders [86] and are often used in addition to or as replacement for artificial tears. The drops contain growth factors, fibronectin, and vitamins, and support proliferation, migration, and differentiation of the corneal epithelium [87]. They are produced by centrifugation of the patient's own peripheral blood followed by dilution in sterile physiological saline [88]. There is variation in the methods of serum drop preparation, and neither production methods nor application is standardized so far [88].

Theoretically, there is a risk of bacterial contamination during the production process as well as during application of the drops. Sterile manufacturing conditions and proper application are therefore a high priority. If patients are not suitable for venesection, allogeneic serum drops can be an alternative, but with an increase in risk of diseases transmission [89, 90]. In the literature, the number of complications in patients receiving serum eye drops is small and most authors report no complications [88]. Nine clinical studies have used serum drops postoperatively, all of them of autologous origin [9, 12, 26, 27, 29, 30, 32, 34, 35]. Infectious complications were mentioned in only one study using autologous serum. However, it was not possible to attribute this to the drops rather than other facets of the clinical trial [34].

Anti-VEGF agents target angiogenesis at molecular level and have a demonstrated efficacy in reducing corneal neovascularization [91]. Bevacizumab (Avastin) is a full-length, humanized murine monoclonal antibody that recognises all isoforms of VEGF-A [92] and is used off-label in ocular surface pathology. Bevacizumab is relatively safe and well tolerated, but prolonged VEGF blockade may impair wound healing and corneal nerve regeneration. Therefore, care should be taken in patients with epithelial defects and neurotrohpic keratopathy [93]. In two papers, bevacizumab was prescribed following CLET when revascularisation recurred [12, 32]. No complications related to this treatment were recorded.

### 6.2. Systemic

Systemic immunosuppression and steroids are used in allogeneic transplantations in order to prevent graft rejection [94, 95]. Although effectively used, these medications are not without safety concerns, since they both can pose risks of mild to severe side effects. The earliest techniques of allogeneic limbal stem cell transplantation required life-long immunosuppressive treatment. The newer allogenic CLET surgery typically requires a shorter period of systemic immunosuppression.

As the volume of transplanted tissue is smaller and allogeneic DNA material was not reported to be present after 9 months following transplantation [2–4], systemic immunosuppression is usually not given longer than 1 year following transplantation. The optimum dose and duration of systemic immunosuppression is not yet defined. In the literature, 14 trials reported the use of systemic steroids [10, 13, 14, 22, 24–31, 33, 35], of which 9 included allogeneic transplantations [25–31, 33, 35]. Immunosuppression was used in 11 trials [20, 22, 25, 28–32, 34–36]. Kawashima et al. used immunosuppression in both allogeneic and autologous graft transplants. Where duration of immunosuppression was reported, it varied from 1 to 18 months, and in the case of steroids, this varied from 2 weeks to 3 months postoperatively. Modifications of the drugs were described due to side effects, but no major complications were noted.

## 7. Marketing Approved ATMP

Holoclar (Chiesi Farmaceutici SpA) is a marketing-approved advanced therapy medicinal product (ATMP) containing autologous limbal stem cells. In February 2015, Holoclar was approved by the European Medicines Agency (EMA) for use in the EU. Since its approval was based on results of retrospective data, the product has been authorised under “conditional approval.” Annual renewal of approval will be guided by results of a currently ongoing multicenter, prospective phase IV clinical trial.

Holoclar is intended for autologous use in adults with moderate or severe LSCD caused by physical or chemical ocular burns. The cells are expanded in cell culture on a fibrin layer with use of lethally irradiated 3T3-J2 feeder layer and FBS [13].

Success ratio of this medicinal product, based on the HLSTM01 study (a multicentre, case-series, noncontrolled, retrospective cohort study in 106 patients) is 72,1%, and no adverse events related to the cells or their culture components were reported [96].

In Europe, ATMPs are strictly regulated by the EMA, which uses specially tailored rules to guarantee a high level of health protection as well as to facilitate market access. Detailed guidelines are in use related to the post authorisation follow-up of efficacy and adverse reactions and risk management [97].

Although authority-applied guidelines help ascertain safe and viable cell products, application procedures are complex and expensive. This translates into a low number of market authorisation applications and restrictions to the public. Strategies should be rethought in order to provide a viable and safe strategy on the market.

## 8. Good Manufacturing Practice

Good manufacturing practice (GMP) provides guidelines for ensuring that biological products are manufactured consistently under quality standards and guarantees that the end product is as safe as possible for the patient. GMP guidance is provided by regulatory bodies around the world. In Europe, this is conducted by the EMA, whilst the Food and Drug Administration (FDA) regulates the US market. Other countries adhere to the GMP guidelines provided by the World Health Organization. European Union law requires that medicinal products for tissue regeneration, including CLET grafts, are produced only by accredited tissue banks under these conditions. Only few centers worldwide are known to produce limbal stem cell grafts under GMP conditions. Only 8 groups have specifically referred to GMP guidelines [9–11, 13, 24, 31, 35, 36] Recently, Sheth-Shah et al. published a review on regulatory requirements in the GMP production of epithelial cell grafts for ocular surface reconstruction. They provided principles of design, construction, validation, and manufacturing within a good manufacturing practice, based on their own experience [98].

## 9. Discussion

The current generation of CLET protocols relies on the use of animal and/or human donor material, all of which carry varying levels of risks for the patient. The use of autologous cells during the CLET procedure avoids risks of graft rejection or immunoreaction. Determining the risks of rejection in allogenic CLET can be complicated since signs of rejection in limbal epithelial stem cell grafts are not clearly defined or detectable. It is therefore likely that rejection rates are underestimated.

If complications are mediated by immunologic reactions, cadaveric CLETs should be more likely to reject than those of living relatives. HLA matching could theoretically reduce the complication rate, but this is yet to be proven. Almost all of the reported trials rely on the use of animal-derived culture material, such as 3T3 and FBS. Although there were no reported side effects related to the use of these products, their use carries theoretically an additional risk of transmitting xenogenic diseases. Human clinical trials should be encouraged to avoid the use of animal and, if possible, human-derived products to minimize the associated health risks for the recipient.

The surgical technique of the biopsy and the CLET transplantation is similarly evolving. Iatrogenic damage to the donor site is very limited but smaller biopsies would be preferable. Higher resolution imaging of the limbus may allow smaller, more targeted biopsies and limit collateral damage to the rest of the limbal cell population. During transplantation, a superficial keratectomy is performed, which has limited safety concerns as long as there is a sufficient residual corneal thickness postoperatively. Determining how much pannus and vascularized cornea removal is necessary depends on the skill and experience of the surgeon. Intraoperative OCT imaging can assist the surgeon in the removal of the fibrovascular pannus. This augments the chances of sparing the viable corneal stroma and can reduce the risk of extreme thinning, iatrogenic ectasia, and wound dehiscence while providing a smooth recipient surface for the composite graft [76].

Postoperative treatment strategies vary widely, and their impact on graft viability is unsure. Autologous serum carries a risk of bacterial contamination during the production process, but in general the advantages are thought to surpass the risks. GMP guidelines also help ensure the safety of the graft products. These principles should be implemented in all future trials. Most papers report low rates of complications and none directly related to the culture process. Attributing a complication to a specific product or even to a surgical technique is not always possible. They can be related to culture products, surgical approaches, stabilization methods, postoperative strategies, or the natural course of the disease. It is therefore difficult to determine the optimal and safest manufacturing protocol. The limited patient numbers and variable amounts of available information further limit our ability to make general evidence-based guidelines.

## 10. Conclusion

With the implementation xenogenic free protocols, CLET surgery can offer a safe and effective treatment modality in LSCD. Throughout the whole CLET process, strict guidelines for good manufacturing practice, quality control, and documentation must be established and maintained prior, in order to guarantee optimal safety. The perfect CLET protocol would include an animal and allogeneic human tissue-free culture method governed by the GMP principles.

## Figures and Tables

**Table 1:** Culture techniques of the included trial reports.

Author	Year	Eyes transplanted (*n*)	Autologous/allogeneic	Scaffold	3T3 (yes/no)	Serum	Duration of culture (days)	Animal-free culture conditions	GMP conditions
Sangwan et al. [5]	2006	78	Autologous	HAM	No	FBS or AS	10–14	No	No
Nakamura et al. [28]	2006	9	Allogenic (*n* = 7), autologous (*n* = 2)	HAM	Yes (allo), no (auto)	AS	15-16	Yes	No
Ang et al. [25]	2007	1	Allogenic	HAM	Yes	FS	Up to 28	No	No
Fatima et al. [6]	2007	1	Autologous	HAM	No	AS	Approx 14	No	No
Kawashima et al. [29]	2007	6	Autologous (*n* = 2), allogenic (*n* = 4)	HAM	Yes	FS or AS	Approx 21	No	No
Shimazaki et al. [30]	2007	27	Autologous (*n* = 7), allogenic (*n* = 20)	HAM	No (*n* = 16), yes (*n* = 11)	AS	Approx 14–21	No	No
Shortt et al. [31]	2008	10	Autologous (*n* = 3), allogenic (*n* = 7)	HAM	No	FS	14–21	No	Yes
Satake et al. [7]	2009	1	Autologous	HAM	Yes	AS	14	No	No
Di Girolamo et al. [8]	2009	2	Autologous	Silixane hydrogel CL	No	AS	10	Yes	No
Meller et al. [26]	2009	1	Allogenic	HAM	No	AS	X	No	No
Pauklin et al. [32]	2010	44	Autologous (*n* = 30), allogenic (*n* = 14)	HAM	No	AS	Approx 14	No	No
Kolli et al. [9]	2010	8	Autologous	HAM	No	AS	10–14	Yes	Yes
Gisoldi et al. [10]	2010	6	Autologous	Fibrin	Yes	X	14–16 days	No	Yes
Di Iorio et al. [11]	2010	166	Autologous	Fibrin	Yes	FBS	X	No	Yes
Thanos et al. [12]	2010	1	Autologous	HAM	No	AS	X	No	No
Rama et al. [13]	2010	107	Autologous	Fibrin	Yes	FBS	14–16	No	Yes
Baradaran-Rafii et al. [14]	2010	8	Autologous	HAM (denuded)	No	FBS	10–14	No	No
Sangwan et al. [15]	2011	200	Autologous	HAM	No	AS	10–14	Yes	No
Sharma et al. [33]	2011	50	Autologous (*n* = 34), allogenic (*n* = 16)	HAM	No	FBS	21	No	No
Basu et al. [16]	2012	50	Autologous	HAM	No	AS	10–14	Yes	No
Prabhasawat et al. [4]	2012	19	Autologous (*n* = 12), allogenic (*n* = 7)	HAM denuded	No	AS	15–23	X	No
Pellegrini et al. [24]	2013	157	Autologous	Fibrin	Yes	FBS	Approx 15	No	Yes
Sejpal et al. [17]	2013	107	Autologous	HAM	No	FBS or AS	10–14	No	No
Pathak et al. [18]	2013	9	Autologous	HAM	No	AS	14–21	Yes	No
Qi et al. [27]	2013	42	Allogenic	HAM	Yes	FBS	X	No	No
Subramaniam et al. [19]	2013	40	Autologous	HAM	No	AS	10–15	No	No
Sharma et al. [20]	2013	4	Autologous	HAM	No	FBS	14	No	No
Vazirani et al. [21]	2014	70	Autologous	HAM	No	FBS or AS	10–14	No	No
Zakaria et al. [35]	2014	18	Autologous (*n* = 15), allogenic (*n* = 3)	HAM	No	AS	14	Yes	Yes
Ramírez et al. [36]	2015	20	Autologous (*n* = 11), allogenic (*n* = 9)	HAM	No	FBS	7–14	Yes	Yes
Bobba et al. [22]	2015	7	Autologous	Silixane hydrogel CL	No	AS	9–16	Yes	No
Pedrotti et al. [23]	2015	13	Autologous	Fibrin	Yes	FBS	Approx 14	No	No

AS: autologous serum; FBS: fetal bovine serum; CL: contact lens; HAM: human amniotic membrane; 3T3: 3T3 feeder layer; GMP: good manufacturing practice; Approx: approximately.
